# An Investigation of the Anticancer Mechanism of *Caesalpinia sappan* L. Extract Against Colorectal Cancer by Integrating a Network Pharmacological Analysis and Experimental Validation

**DOI:** 10.3390/plants14020263

**Published:** 2025-01-18

**Authors:** Mibae Jeong, Jaemoo Chun, Sang-Min Park, Heerim Yeo, Se Won Na, In Jin Ha, Bonglee Kim, Mi-Kyung Jeong

**Affiliations:** 1 KM Convergence Research Division, Korea Institute of Oriental Medicine, Daejeon 34054, Republic of Korea; 2 KIOM School, University of Science & Technology (UST), Daejeon 34054, Republic of Korea; 3 College of Pharmacy, Chungnam National University, Daejeon 34134, Republic of Korea; 4 Korean Medicine Clinical Trial Center (K-CTC), Korean Medicine Hospital, Kyung Hee University, Seoul 02447, Republic of Korea; 5 Department of Pathology, College of Korean Medicine, Kyung Hee University, Seoul 02447, Republic of Korea

**Keywords:** *Caesalpinia sappan* L., colorectal cancer, network pharmacology, apoptosis, MC38 tumor

## Abstract

*Caesalpinia sappan* L. has exhibited various pharmacological effects, yet its anticancer activities against colorectal cancer (CRC) and underlying molecular mechanisms remain unclear. This study investigated the anticancer properties of an ethanol extract of *C. sappan* L. (CSE) against CRC cells, focusing on the identification of bioactive compounds and their mechanisms of action. A network pharmacology analysis was conducted to identify potential CRC targets and bioactive compounds of CSE, using LC-MS for compound identification. The anticancer effects of CSE were then validated through in vitro and in vivo models of CRC. The network pharmacological approach identified 87 overlapping genes between CSE targets and CRC-related genes, with protein–protein interaction analysis highlighting 33 key target genes. CSE inhibited cell proliferation in human CRC cell lines, including HCT 116, KM12SM, HT-29, and COLO 205, and induced apoptosis via caspase 3/7 activation. Western blot analyses confirmed the modulation of critical signaling pathways, including STAT3, AKT, and mitogen-activated protein kinases. Furthermore, CSE significantly suppressed tumor growth in MC38 CRC-bearing mice. These findings suggest that CSE possesses substantial potential as a natural anticancer agent for CRC treatment, highlighting the need for further exploration in therapeutic development.

## 1. Introduction

Colorectal cancer (CRC) is one of the most prevalent malignancies globally and the second leading cause of cancer-related deaths with significant morbidity and death rates [1]. Current therapeutic strategies, including surgery, chemotherapy, and radiation, often lead to side effects and are insufficient in preventing recurrence [2]. Additionally, the increasing incidence of chemoresistance and tumor heterogeneity further complicates treatment outcomes, highlighting the urgent need for novel therapeutic approaches that can offer higher efficacy and better patient outcomes with fewer adverse effects [3]. This has prompted a search for alternative treatments, particularly those derived from natural products, which are known for their diverse biological activities and lower toxicity with minimal or no side effects [4].

*Caesalpinia sappan* L., a traditional medicinal plant, has been widely used for its therapeutic properties, including anti-inflammatory [5], antioxidant [6], and antimicrobial [7] effects. Its active components, such as brazilin, sappanone, and protosappanin, have been extensively studied for their pharmacological effects, with brazilin being the most prominent for its various bioactivities. Moreover, recent studies have demonstrated that *C. sappan* L. extract possesses significant anticancer properties [8,9,10]. However, specific research on its effects on CRC remains limited, and further studies are needed to explore its specific targets in CRC.

Network pharmacology, an emerging field that integrates systems biology, bioinformatics, and pharmacology, enables a deeper understanding of the interactions between drugs and biological systems [11]. This approach considers the multi-target nature of drugs, especially those derived from natural sources, and maps out the complex networks of interactions between compounds and their molecular targets [12]. Moreover, the integration of LC-MS and network pharmacology can provide a powerful approach to dissecting the pharmacological effects of natural products systematically. This involves a comprehensive approach combining LC-MS analysis to pinpoint the active constituents and network pharmacology to unravel the complex interactions and pathways these compounds influence [13]. By identifying the active components and their targets, this approach allows us to gain insights into the complex mechanisms through which natural products exert their anticancer effects, paving the way for the development of novel therapeutic agents. This view helps in understanding the synergistic effects of multiple compounds and their impact on various biological pathways and processes involved in disease mechanisms [14].

This study aims to explore the anticancer potential of *C. sappan* L. extract against CRC through advanced analytical techniques and computational methods. We provide a qualitative assessment of the constituents using a metabolomic profiling approach, in addition to network pharmacology and experimental validation. The findings could significantly contribute to the development of new, natural-based therapies for CRC, providing safer and more effective treatment options.

## 2. Results

### 2.1. Identification of Chemical Components in Ethanol Extract of C. sappan L. (CSE)

**Figure 1:**
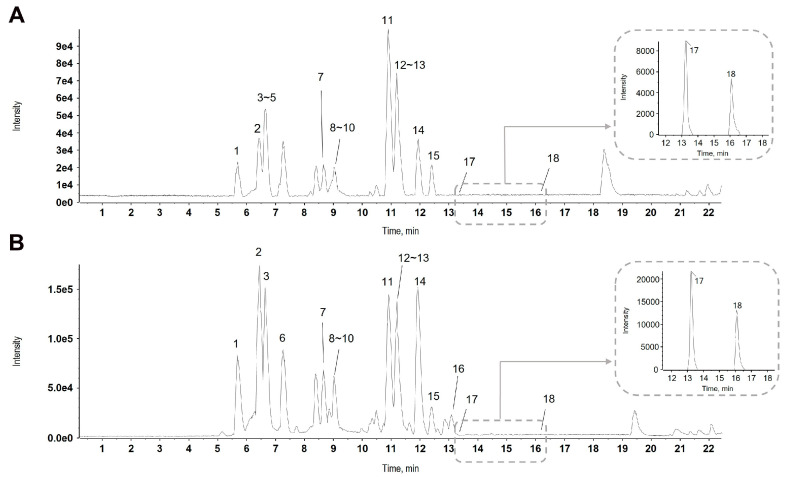
The representative base peak chromatogram (BPC) of the ethanol extract of *Caesalpinia sappan* L. (CSE) was obtained using LC-ESI-QTOF MS/MS analysis in positive (**A**) and negative (**B**) ion modes. 1: 3′-O-methylcatechin, 2: brazilin, 3: protosappanin B, 4: chrysophanol, 5: dihydroxyxanthone, 6: 4′-O-methylcatechin, 7: lyoniresinol, 8: 3′-O-methylbrazilin, 9: brazilein, 10: sappanone B, 11: sappanchalcone, 12: 5,7,4′-tri-O-methylcatechin, 13: sappanone A, 14: 5,7,3′-tri-O-methyl (-)-epicatechin, 15: brazilane, 16: 10,11-dihydroxydracaenone C, 17: 4′-O-methylbrazilin, and 18: tricin. The extracted ion chromatograms of compounds 17 and 18 are shown above each BPC.

The total ion chromatograms of CSE were obtained by ultrahigh-performance liquid chromatography (UHPLC)-QTOF MS, and the base peak chromatograms were generated to plot the intensity of the largest peak in each scan as a function of retention time while minimizing artifacts caused by noisy background peaks. The molecular formula was then generated based on the chromatographic peak information using PeakView 2.2 and MasterView software (SCIEX, Foster City, CA, USA). The expected compounds contained in CSE were preliminarily identified by comparison with the relevant literature on the chemical constituents [15,16,17,18]. Next, the corresponding fragment ions of each compound were compared with the MS/MS spectra of reference standards or an in-house MS/MS library, as well as online databases such as Global Natural Product Social Molecular Networking (GNPS), MASS bank, or Metlin (Figure 1A,B). As a result, eighteen compounds were tentatively identified in CSE by comparing them with mass spectrometry and retrieving the reference literature. These eighteen compounds included 3′-O-methylcatechin, brazilin, protosappanin B, chrysophanol, dihydroxyxanthone, 4′-O-methylcatechin, lyoniresinol, 3′-O-methylbrazilin, brazilein, sappanone B, sappanchalcone, 5,7,4′-tri-O-methylcatechin, sappanone A, 5,7,3′-tri-O-methyl (-)-epicatechin, brazilane, 10,11-dihydroxydracaenone C, 4′-O-methylbrazilin, and tricin as shown in Table 1 with detailed chromatographic and mass spectroscopic data by UHPLC-QTOF MS/MS-based chemical profile analysis. The observed MS/MS spectra of the eighteen compounds are provided in Figure S1.

### 2.2. Network Pharmacology Analysis Predicts the Target Pathways of CSE in CRC

**Figure 2:**
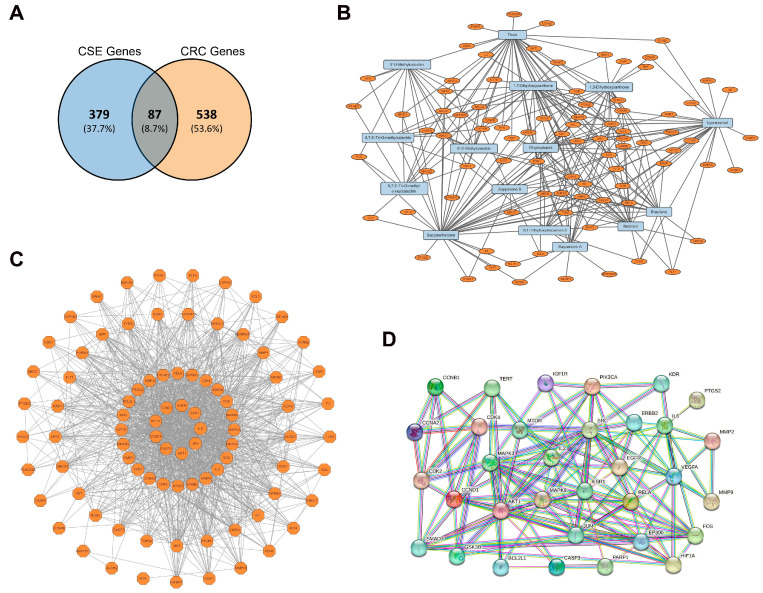
Network pharmacological analysis of *C. sappan* L. (**A**) Venn diagram for 466 target genes of CSE and 625 CRC-related genes. Eighty-seven genes were common. (**B**) Network of compounds and target genes of CSE. Each node represents either a compound or a target gene, and the edges represent interactions between these compounds and genes. (**C**) Protein–protein interaction (PPI) of the common 87 target genes. The 33 genes in the inner circle have more or stronger connections with other genes. (**D**) STRING analysis of the PPI network of selected 33 target genes.

To explore the potential molecular targets of CSE, a network pharmacological analysis was conducted. A total of 466 target genes related to CSE were obtained from two databases, Similarity Ensemble Approach (SEA) and Swiss Target Prediction (STP). Simultaneously, 625 CRC-related genes were retrieved from GeneCards, DisGeNet, and the Comparative Toxicogenomics Database (CTD) using specific relevance score thresholds. The intersection of these two datasets identified 87 common target genes, as shown in the Venn diagram (Figure 2A). A compound–target gene interaction network was constructed using Cytoscape 3.10.2, linking the compounds identified in CSE to the 87 common target genes (Figure 2B and Figure S2A). This network provides insight into the relationships between the bioactive compounds of *C. sappan* L. and their associated molecular targets in CRC, suggesting potential multi-target therapeutic mechanisms. The STRING database was employed to construct a protein–protein interaction (PPI) network for the 87 common target genes. This network analysis identified key interactions between target proteins, highlighting potential hubs that may play significant roles in CRC-related pathways. The 9 central genes are highly interconnected, surrounded by 24 additional genes in the next layer of the network, which likely represent key regulatory hubs involved in CRC-related pathways (Figure 2C and Figure S2B). Furthermore, STRING analysis of the PPI network allowed for the selection of 33 core target genes with significant relevance to CRC (Figure 2D).

### 2.3. The Identification of Core Genes and Pathway Enrichment Analysis Reveal Therapeutic Mechanisms in CSE for Cancer Progression

**Figure 3:**
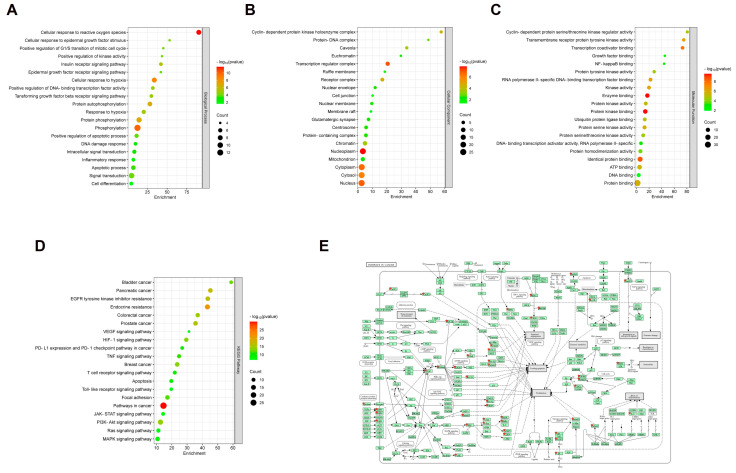
Pathway enrichment analysis for the 33 target genes of *C. sappan* L. (**A**) The Gene Ontology (GO) biological process (BP) gene sets, (**B**) GO cellular component (CC) gene sets, (**C**) GO molecular function (MF) gene sets, and (**D**) the Kyoto Encyclopedia of Genes and Genomes (KEGG) gene sets. (**E**) Pathways in cancer involved in cell cycle regulation, apoptosis, and signal transduction.

The core genes were selected based on their centrality and potential involvement in key molecular pathways related to cancer progression and therapeutic response. Pathway enrichment analysis was performed for the 33 selected core target genes using the DAVID bioinformatics tool. The analysis identified several enriched Gene Ontology (GO) terms, including biological processes (BPs), cellular components (CCs), and molecular functions (MFs), alongside pathways in the Kyoto Encyclopedia of Genes and Genomes (KEGG). In the BP analysis, significant terms were fundamental to the control of cancer cell growth and survival (Figure 3A). In CC and MF analysis, enriched terms emphasized the involvement of these core genes in critical subcellular structures and complexes in signaling and transcription regulation, both of which are essential for cancer-related pathways (Figure 3B,C). Additionally, KEGG pathway enrichment underscored the relevance of the AKT, mitogen-activated protein kinases (MAPKs), VEGF, and STAT3 signaling pathways, which are known to regulate cell survival, proliferation, and apoptosis (Figure 3D). The enriched pathways collectively indicated that the therapeutic effects of CSE could be mediated by the modulation of multiple interconnected pathways involved in cell cycle regulation, apoptosis, and signal transduction (Figure 3E and Figure S3). These findings suggest that the therapeutic effects of CSE may be mediated through the modulation of these critical biological processes and pathways.

### 2.4. CSE Inhibited the Cell Proliferation of the Human CRC Cell Lines

**Figure 4:**
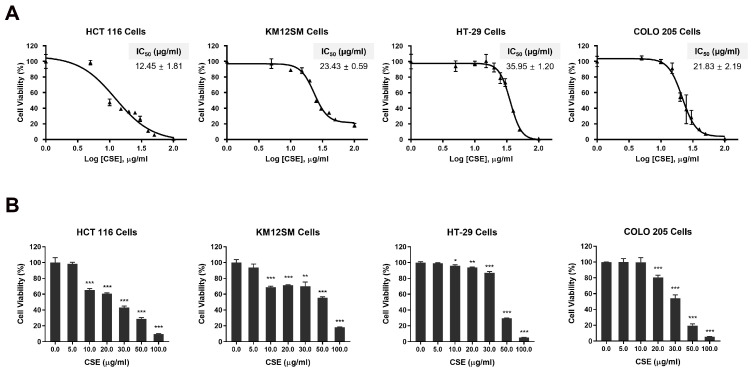
Inhibitory effect of CSE on the cell proliferation of human CRC cell lines. (**A**) The determination of ATP levels in HCT 116, KM12SM, HT-29, and COLO 205 cells. Cells were treated with CSE (0–100 μg/mL) for 48 h. ATP levels were assessed using CellTiter-Glo 2.0. The dose–response curves and half-maximal inhibitory concentration (IC_50_) values are determined. (**B**) The effect of CSE on cell proliferation of CRC cells. Cells were treated with CSE (0–100 μg/mL) for 48 h. Cell viability was assessed through a Cell Counting Kit-8 assay (CCK-8). * *p* < 0.05, ** *p* < 0.01, and *** *p* < 0.001, compared to the control (0 μg/mL) group.

To investigate the inhibitory effect of CSE on the proliferation in CRC cells, the cell viability of HCT 116, KM12SM, HT-29, and COLO 205 cells was assessed. HCT 116 and COLO 205 cells exhibited greater cytotoxicity compared to KM12SM and HT-29 cells, with significant differences in their half-maximal inhibitory concentration (IC_50_) values (Figure 4A). We also confirmed that CSE effectively inhibited cell proliferation in HCT 116 and COLO 205, as indicated by a concentration-dependent decrease in mitochondrial dehydrogenase activity (Figure 4B). These results suggest that CSE effectively suppresses CRC cell growth.

### 2.5. CSE Induced Caspase-Mediated Apoptosis of CRC Cells

**Figure 5:**
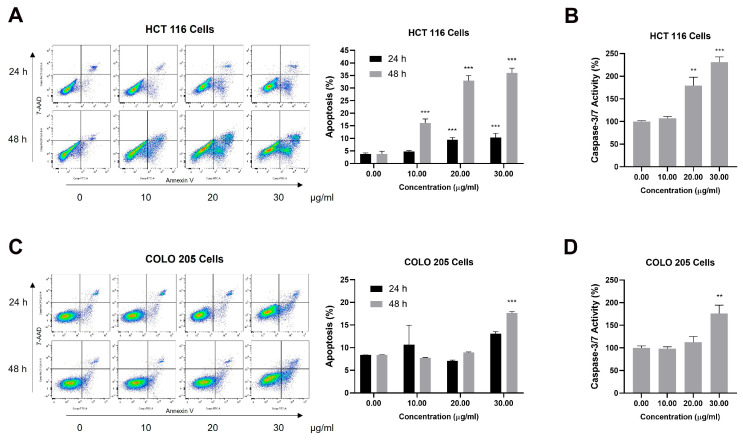
Effect of CSE on apoptosis induction in HCT 116 and COLO 205 cells. (**A**) Flow cytometric analysis and apoptosis percentage of HCT 116 cells stained with Annexin V/7-AAD after 24 h and 48 h of CSE treatment. The apoptotic rate means the rate of Annexin V-positive cells. (**B**) The caspase 3/7 activity of HCT 116 cells was evaluated using the Caspase-Glo 3/7 Assay Kit after 48 h of CSE treatment. (**C**) Flow cytometric analysis and apoptosis percentage of COLO 205 cells stained with Annexin V/7-AAD after 24 h and 48 h of CSE treatment. The apoptotic rate means the rate of Annexin V-positive cells. (**D**) The caspase 3/7 activity of COLO 205 was evaluated using the Caspase-Glo 3/7 Assay Kit after 48 h of CSE treatment. Bars represent the mean ± SD (n = 3). ** *p* < 0.01 and *** *p* < 0.001, compared to the control (0 μg/mL) group.

Annexin V/7-AAD double staining was performed to detect apoptosis in HCT 116 and COLO 205. The cells were treated with concentrations of 0, 10, 20, and 30 μg/mL for 24 h and 48 h. After the exposure, a significant increase in early and late apoptosis was observed in both cells. We also found that the percentage of apoptosis increased in a dose- and time-dependent manner in both HCT 116 and COLO 205 cells (Figure 5A,C). To further confirm the apoptotic process induced by CSE, caspase-3/7 activity was measured using the Caspase-Glo 3/7 Assay Kit. After 48 h of treatment, the percentage of apoptosis at 10 μg/mL of CSE was comparable. However, 30 μg/mL of CSE treatment indicated significantly elevated activity in HCT 116 and COLO 205 (Figure 5B,D). These results suggest that CSE could promote caspase-dependent apoptosis in CRC cells.

### 2.6. CSE Regulated the Expression of Apoptosis- and Cell-Cycle-Related Proteins

**Figure 6:**
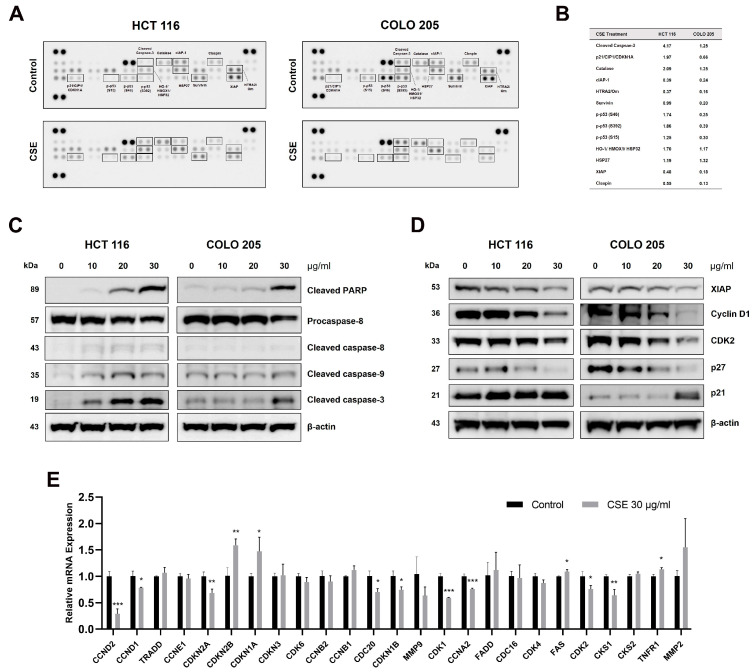
Effect of CSE on apoptosis and cell cycle regulatory proteins in HCT 116 and COLO 205 cells. (**A**) Images of protein array showing the expression of apoptosis-related proteins in control and CSE-treated HCT 116 and COLO 205 cells. Cells were treated with 30 μg/mL CSE for 48 h. (**B**) Quantitative comparison of selected protein levels, shown as average fold-difference relative to control (0 μg/mL), based on duplicates from the protein array. (**C**) The expression levels of apoptotic proteins, including PARP, caspase-8, caspase-9, and caspase-3. (**D**) The expression levels of cell cycle regulatory proteins, including XIAP, cyclin D1, CDK2, p27, and p21. (**E**) Relative mRNA expression of cell-cycle-related genes in control and CSE-treated HCT 116 cells. Bars represent the mean ± SD (n = 3). * *p* < 0.05, ** *p* < 0.01, and *** *p* < 0.001, compared to the control group.

To investigate the molecular mechanism of CSE-induced apoptosis, we used an apoptosis array. HCT 116 and COLO 205 cells were treated with 30 μg/mL of CSE for 48 h. We confirmed that several apoptosis-related proteins were regulated by the treatment of CSE. The treatment of CSE increased the expression levels of cleaved caspase-3, HO-1, catalase, and HSP27 but decreased the expression of cIAP-1, claspin, HTRA2, and XIAP in both HCT 116 and COLO 205 cells (Figure 6A,B). Next, we confirmed that the expression levels of cleaved PARP, caspase-8, caspase-9, and caspase-3 were elevated in both HCT 116 and COLO 205 cells by CSE treatment, indicating the induction of apoptosis in these cell lines (Figure 6C). Moreover, CSE treatment led to alterations in the expression of several key cell cycle regulatory proteins. Specifically, the levels of XIAP, cyclin D1, CDK2, and p27 were significantly decreased, whereas the level of p21 was elevated (Figure 6D). These findings suggest that CSE treatment may inhibit cell cycle progression by modulating proteins involved in cell cycle checkpoints and apoptosis inhibition. The relative mRNA expression of cell-cycle-related genes in HCT 116 cells was also assessed. Consistent with the protein expression results, CSE treatment significantly reduced the mRNA expression of genes promoting cell cycle progression (e.g., *CCND2* and *CDK2*). In contrast, genes involved in cell cycle arrest (e.g., *CDKN1A* and *CDKN2B*) were upregulated (Figure 6E). These transcriptional changes further support the hypothesis that CSE treatment interferes with cell cycle progression and promotes apoptosis in CRC cells.

### 2.7. CSE Inhibited the Phosphorylation of STAT3 and Triggered a Cellular Stress Response

**Figure 7:**
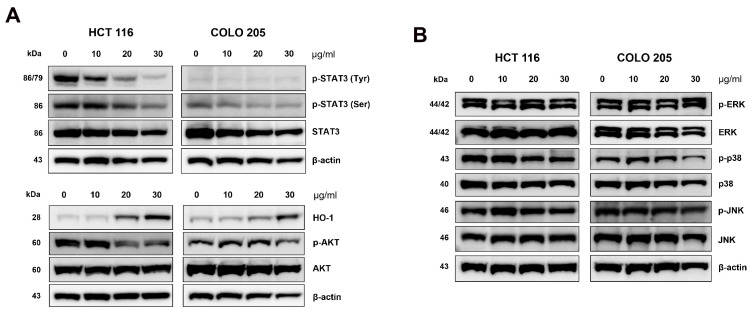
Effect of CSE on STAT3, AKT, and mitogen-activated protein kinase (MAPK) pathways in HCT 116 and COLO 205 cells. (**A**) Protein expression of STAT3 and AKT pathways. (**B**) Protein expression of MAPK pathways, such as ERK, p38, and JNK.

To explore the upstream regulatory mechanisms involved in apoptosis and cell cycle regulation, we examined the STAT3, AKT, and MAPK signaling pathways. As shown in Figure 7A, CSE treatment significantly suppressed both tyrosine and serine phosphorylation of STAT3 in HCT 116, whereas only serine phosphorylation was reduced in COLO 205 cells. Additionally, the AKT pathway showed reduced phosphorylation by CSE treatment, implying that CSE may inhibit cell survival signaling to promote apoptosis or cell cycle arrest. Interestingly, CSE upregulated the expression of HO-1, indicating that CSE triggers a cellular stress response. In contrast, CSE treatment had no significant effect on the MAPK pathways, as evidenced by minimal changes in the phosphorylation of ERK, p38, and JNK (Figure 7B). Overall, these findings suggest that CSE modulates apoptosis and cell cycle regulation by influencing the STAT3 and AKT pathways, potentially promoting anticancer effects in CRC cells through a combination of signaling alterations and stress induction.

### 2.8. CSE Modulated Cancer-Related Inflammation and Immunity

**Figure 8:**
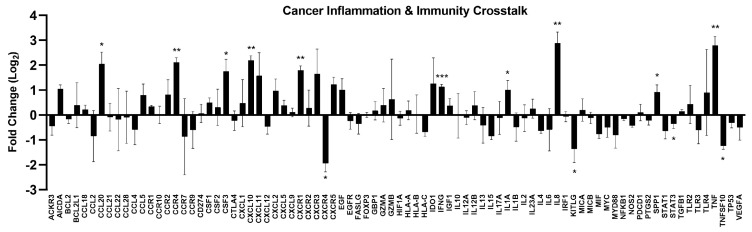
Fold change in gene expression related to cancer inflammation and immunity crosstalk in CSE-treated HCT 116 cells. Cells were treated with CSE (30 μg/mL) for 24 h. Fold change (Log_2_) in expression levels of genes following CSE treatment was calculated relative to control (0 μg/mL) cells. Bars represent the mean ± SD of three independent experiments. * *p* < 0.05, ** *p* < 0.01, and *** *p* < 0.001, compared to the control group.

Cancer immunity plays a critical role in controlling tumor growth and progression by influencing the inflammatory responses and immune signaling pathways within the tumor microenvironment. Therefore, we investigated the altered expression related to cancer inflammation and immunity crosstalk in CSE-treated HCT 116 cells. As shown in Figure 8, treatment with 30 µg/mL CSE showed significant alterations of several genes associated with inflammatory responses and immune regulation. Notably, genes such as *CCL20*, *CCR4*, *CSF3*, *CXCL10*, *CXCR1*, *IFNG*, *IL1A*, *IL8*, *SPP1*, and *TNF* were upregulated, indicating enhanced antigen presentation and immune signaling. Conversely, some genes, including *CXCR4*, *KITLG*, *STAT3*, and *TNFSF10*, were downregulated, suggesting a potential suppression of specific pathways related to tumor proliferation and vascular development. The differential expression pattern observed highlights the complex role of CSE in modulating genes involved in cancer-related inflammation and immune response, potentially contributing to its anticancer effects by altering the tumor microenvironment and immune interactions.

### 2.9. CSE Suppressed the Tumor Growth of the MC38 Colorectal Tumor

**Figure 9:**
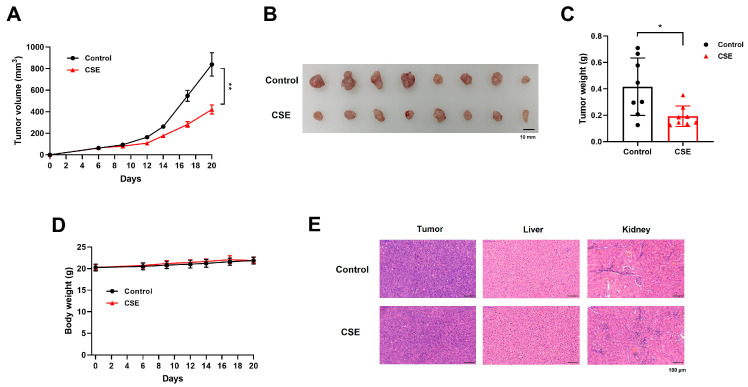
CSE suppressed the tumor growth of the MC38 colorectal tumor. MC38 cells (1 × 10^5^ cells) were inoculated subcutaneously into the right flank of C57BL/6 female mice. Mice were divided into the control and CSE-treated groups (n = 8, each group). One week after injection, CSE was administered daily at a dose of 100 mg/kg via oral gavage, with 1% CMC in DW used as a control. (**A**) Tumor volumes were measured every 3 days after the treatment. (**B**) Tumor images from mice. Scale bar represents 10 mm. (**C**) Tumor weights were shown. (**D**) Body weight changes of MC38-bearing mice. (**E**) Hematoxylin and eosin staining of tumor, liver, and kidney. Scale bar 100 µm. * *p* < 0.05 and ** *p* < 0.01, compared to the control group.

MC38 cells (1 × 10⁵) were inoculated subcutaneously into the right flank of C57BL/6 mice, which were then divided into control and CSE-treated groups (n = 8 per group). One week after tumor inoculation, when the average tumor volume reached approximately 60 mm^3^, daily administration of CSE (100 mg/kg) was initiated, with 1% carboxymethyl cellulose (CMC) in distilled water (DW) used as a control. CSE treatment resulted in a marked reduction in tumor size compared to the control group (Figure 9A,B). Tumor weights were significantly lower in the CSE-treated group (Figure 9C). Notably, there were no significant changes in body weight between the two groups, indicating that CSE did not induce major systemic toxicity (Figure 9D). In addition, histopathological analysis demonstrated that there were no significant signs of tissue damage or toxicity in the liver and kidney of CSE-treated mice (Figure 9E). These results suggest that CSE effectively inhibits colorectal tumor growth without causing adverse side effects.

### 2.10. CSE Regulated Cell Proliferation, Apoptosis, and Immune Pathways in Cellular Processes

**Figure 10:**
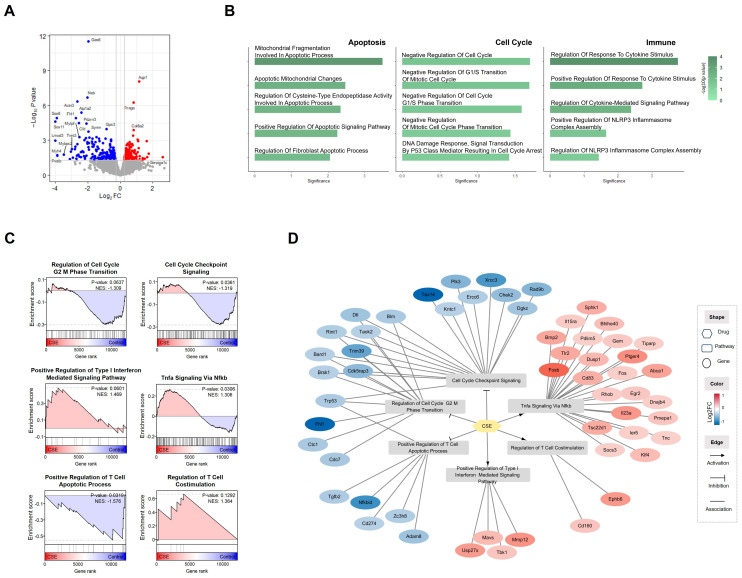
Transcriptomic effects of CSE treatment. (**A**) Volcano plot depicting differentially expressed genes (DEGs) between CSE-treated and control groups. Red points indicate significantly upregulated genes, while blue points represent significantly downregulated genes (|log_2_ fold change (FC)| > 1.2, *p*-value < 0.05). (**B**) EnrichR analysis showing significantly enriched cellular processes. Key processes include apoptosis, cell cycle regulation, and immune response, based on the DEGs from the CSE treatment group. (**C**) Gene Set Enrichment Analysis (GSEA) plots demonstrating the impact of CSE on specific pathways. Cell cycle checkpoint signaling is significantly influenced, with both upregulation and downregulation observed across the ranked gene list. T-cell signaling pathways, including co-stimulation and regulation of apoptosis, are notably enriched. Pathways related to immune responses, such as interferon-mediated signaling and inflammasome assembly, show significant enrichment. (**D**) Drug–pathway–gene interaction network reconstructed using the GSEA results. The network highlights the interactions between CSE-regulated genes (circles), key pathways (squares), and drugs (hexagons). Node colors represent the log_2_ fold change in gene expression, with connections indicating regulatory relationships: arrows signify positive regulation, T-shaped ends indicate negative regulation, and lines represent associations. Only genes with |FC| > 1.2 are included, aligned with the regulatory patterns of the pathways.

The transcriptome analysis identified significant regulatory effects across several key cellular processes. The volcano plot (Figure 10A and Figure S4A) illustrates the differentially expressed genes (DEGs) between the CSE-treated and control groups, revealing a substantial number of significantly regulated genes. To further investigate, we performed EnrichR analysis using these DEGs (Figure 10B). The results aligned with previous findings, showing strong enrichment in pathways associated with apoptosis, negative regulation of the cell cycle, and immune responses, including cytokine activity. Notably, TNF-α signaling and Type I IFN signaling were prominently activated, suggesting robust immune responses and interferon pathway activation (Figure 10C). We also explored the potential effects on T-cell regulation, given the relevance of these pathways. T-cell-activation-related pathways, such as T-cell co-stimulation, were found to be upregulated. Furthermore, pathways regulating T-cell apoptosis and the G2/M phase transition were altered, indicating the presence of active checkpoint control mechanisms. To better understand the molecular mechanisms through which CSE influences these pathways, we constructed a drug–pathway–gene network (Figure 10D and Figure S4B). The analysis revealed that CSE downregulated genes across three inhibited pathways while upregulating genes across three activated pathways. These complex interactions underscore the multifaceted nature of CSE’s compounds, which modulate various molecular targets involved in its anticancer effects.

## 3. Discussion

Natural products have long been recognized for their therapeutic potential, particularly due to their multi-compound nature, which allows them to target multiple biological pathways simultaneously [19]. This multi-target approach is highly advantageous in treating complex diseases like cancer, where single-target therapies may be limited by drug resistance and side effects. The diversity of bioactive compounds in natural products enables them to modulate various molecular mechanisms, contributing to their effectiveness as anticancer agents [20]. Recently, network pharmacology has experienced rapid advancements. By constructing networks that map the interactions between multiple compounds and their potential targets, network pharmacology enables a comprehensive analysis of how each component contributes to the overall therapeutic effects. This approach is particularly valuable for understanding the complex mechanisms of natural products, as it can reveal the synergistic effects of multiple compounds acting on diverse biological pathways [21,22].

Cancer presents a complex therapeutic challenge, especially CRC, which has high rates of recurrence and resistance to conventional treatments [23]. This complexity underscores the potential value of multi-targeted therapies that can address various aspects of tumor growth, survival, and immune evasion [24]. In this study, we aimed to explore the anticancer effects of CSE against CRC by identifying bioactive compounds and elucidating their mechanisms through a combination of network pharmacology and experimental validation. Our findings demonstrate that CSE exhibits significant anticancer activity, potentially mediated through the modulation of multiple cellular pathways and targets, which supports its potential as a therapeutic agent for CRC. Through network pharmacological analysis, we identified key pathways and targets affected by the major compounds in CSE, providing a mechanistic understanding of its effects. These findings are further corroborated by experimental validation, which highlights the CSE’s capability to inhibit tumor growth and modulate cancer cell signaling. Our network pharmacology analysis identified key molecular pathways potentially modulated by CSE, which guided the selection of specific targets for experimental validation. The experimental results generally supported the predictions, particularly in the inhibition of cell proliferation and the regulation of apoptosis and cell cycle pathways. These findings were consistent with the predicted molecular targets, including PI3K/AKT, STAT3, and MAPKs. However, some discrepancies were observed between the network predictions and experimental results, likely due to the complexity of natural product mixtures and the limitations of network pharmacology in capturing all relevant interactions and targets. Nevertheless, the evidence suggests that CSE, as a multi-target agent, holds promise for overcoming the challenges associated with single-target therapies and improving treatment outcomes for CRC patients.

Our study identified a wide range of bioactive compounds within CSE that contribute to its multi-target anticancer effects. These compounds include flavonoids, lignans, and phenolic compounds, each potentially acting on different molecular targets involved in cancer progression. Brazilin, brazilein, sappanchalcone, and protosappanin B are known to inhibit cell proliferation, targeting cell cycle and apoptosis pathways [25,26,27]. 3′-O-methylcatechin and 4′-O-methylcatechin, as catechin derivatives, could offer antioxidant and anti-inflammatory effects, potentially reducing oxidative stress in cancer cells [28]. Lyoniresinol regulates the Akt/GSK-3β/Nrf2 signaling pathway [29]. These bioactive compounds contribute to the multifaceted anticancer effects observed, which are particularly advantageous in CRC, a disease characterized by its complexity and resistance to conventional treatments. Brazilin, protosappanin B, and sappanchalcone appeared at higher concentrations, whereas 10,11-dihydroxydracaenone C, 4′-O-methylbrazilin, and tricin were detected in lower concentrations. Although these minor compounds are less abundant, the combination of flavonoids, lignans, and phenolic compounds in CSE suggests the potential for synergy commonly observed in natural products, where individual components may enhance each other’s effects. Therefore, we included all identified compounds in the subsequent network pharmacology and pathway analyses to gain a comprehensive understanding of how these compounds interact with various cellular targets and pathways. The observed inhibition of key oncogenic pathways, such as PI3K/AKT, STAT3, and MAPK, aligns with CSE’s demonstrated effects on cancer cell proliferation in our experimental models. Although quantitative analysis of the components in CSE was not performed, we present for the first time a qualitative assessment of its components using a metabolomics profiling approach. This method enabled the identification of key metabolites within the extract, providing a foundational understanding of its chemical composition.

In this study, we investigated the targets identified through network pharmacology for experimental validation and the antitumor potential of CSE in the CRC model. Our results showed that CSE significantly inhibited cell viability and proliferation, mainly by inducing apoptosis. Apoptosis, a programmed cell death mechanism crucial for eliminating cancer cells, plays a critical role in its anticancer mechanism [30]. In addition, CSE altered the expression of cell cycle regulatory proteins. The downregulation of XIAP, cyclin D1, and CDK2, coupled with the upregulation of p21, indicates that CSE might exert its antiproliferative effects by interfering with cell cycle progression. By promoting the expression of p21, CSE could be contributing to cell cycle arrest at the G1/S checkpoint. Additionally, the observed decrease in mRNA levels of cell-cycle-promoting genes, such as *CCND2*, *CDK2*, and *CDK1*, and the increase in cell-cycle-arrest-related genes, like *CDKN1A* and *CDKN2B*, further supports the mechanism by which CSE induces cell cycle arrest. The induction of cell cycle arrest by CSE treatment may ultimately lead to apoptosis. In addition to these findings, we observed that CSE significantly inhibited STAT3 phosphorylation at both tyrosine and serine residues in HCT 116 cells, although its inhibitory effect on tyrosine phosphorylation could not be confirmed in COLO 205 cells. This selective inhibition might be due to the low endogenous levels of STAT3 tyrosine phosphorylation in COLO 205 cells. Given the role of STAT3 in promoting tumor growth and survival [31], its inhibition by CSE highlights a potential mechanism through which CSE exerts its antitumor effects. Furthermore, we examined the effects of CSE on AKT and MAPK signaling pathways. While the MAPK pathways showed minimal changes in response to CSE treatment, the AKT pathway exhibited a decrease in phosphorylation, suggesting that CSE may inhibit cell survival signals mediated by AKT, thereby promoting apoptosis and cell cycle arrest. In addition, the increased expression of HO-1 suggests that CSE induces a cellular stress response. This response could help maintain protein homeostasis under stress, potentially contributing to cancer cell death [32]. However, further research is needed to fully understand the mechanisms by which HO-1 influences apoptosis and cell cycle arrest.

With the growing importance of tumor immune responses and the tumor microenvironment [33], we investigated the expression changes related to cancer inflammation and immunity crosstalk. Cancer immunity is crucial in controlling tumor growth and progression, as it impacts both inflammatory responses and immune signaling pathways within the tumor microenvironment [34]. Although our results exclusively measured the expression of inflammation- and immunity-related genes within the cancer cells, the effects of CSE on cancer-related inflammation and immune responses may contribute to its potential anticancer effects by modifying the tumor microenvironment and immune interactions. Further verification, such as through co-culture systems with immune cells would be required to confirm the involvement of immune cells in CSE’s anticancer effects. Our in vivo analysis confirmed the effectiveness of CSE in reducing tumor progression. This highlights the importance of in vivo validation, as it allows us to observe the therapeutic effects of CSE within a more complex biological environment that closely mimics the tumor microenvironment found in patients [35]. While network pharmacology is a valuable tool for identifying potential targets and pathways, this study has several limitations. Network pharmacology relies on available databases and known molecular interactions, which may not fully capture all potential targets. The complex nature of CSE’s bioactive compounds presents challenges in identifying which specific compounds or combinations are most effective. Although the current analysis emphasizes the potential multi-target therapeutic effects of the extract as a whole, a component-specific network analysis could provide more detailed insights into the roles of individual compounds. Future research should focus on isolating these compounds and examining their individual effects, as well as their potential synergistic interactions, to optimize the therapeutic application of CSE.

## 4. Materials and Methods

### 4.1. Chemicals and Reagents

Rosewell Park Memorial Institute (RPMI) 1640 medium, Dulbecco’s modified Eagle’s medium (DMEM), fetal bovine serum (FBS), and Dulbecco’s phosphate-buffered saline (DPBS) were obtained from Corning (Corning, NY, USA). Trypsin-EDTA solution and penicillin/streptomycin were from Gibco (Grand Island, NY, USA).

### 4.2. Preparation of Plant Extract

*C. sappan* L., collected from Indonesia, was registered as a voucher specimen (Registration Number: DK008) and identified by Prof. Bonglee Kim from Kyung Hee University. The dried and ground heartwood of *C. sappan* L. (200 g) was extracted three times with 2 L of 99% ethanol (Duksan, Gyeonggi-do, Republic of Korea) at a 10:1 (v/w) ratio of 99% ethanol to *C. sappan* L for 1 day. The solution was filtered through Whatman filter paper and evaporated using a rotary evaporator (Eyela, Tokyo, Japan). Finally, the extracted solution was freeze-dried and lyophilized, yielding 1.16 g with an extraction yield of 0.58%.

### 4.3. UHPLC-QTOF MS/MS Analysis for the Chemical Profile of CSE

A UHPLC system (Vanquish, Thermo Fisher Scientific, Sunnyvale, CA, USA) was interfaced with a TripleTOF5600^+^ mass spectrometer (Sciex, Foster City, CA, USA) equipped with a Turbo-V IonSpray. An electrospray ionization source in the negative and positive ion modes was utilized to identify components. The parameters were as follows: mass range, 50–1800 m/z; ion spray voltage, 4.5 kV; source temperature, 550 °C; declustering potential, 50 V; nitrogen, nebulizer gas at 50 L/min; heater gas, 50 L/min; curtain gas, 25 L/min; and collision energy, 10 eV. The gradient conditions for chromatographic separation using 0.1% formic acid in water as eluent A and 0.1% formic acid in acetonitrile as eluent B were as follows: 0–2 min, 5% B; 2–5 min, 5–15% B; 5–12 min, 15–35% B; 12–18 min, 35–50% B; 18–20 min, 50–100% B; 20–25 min, 100% B; and equilibration with 5% B for 4 min at a flow rate of 0.4 mL/min. The temperature of the column was 40 °C, and the auto-sampler was maintained at 4 °C. The injection volume of each sample was 2 μL. The MS/MS data for qualitative analysis were processed to identify and confirm components in CSE.

### 4.4. Network Pharmacology

Target genes of *C. sappan* L. were obtained from two databases: SEA (http://sea.bkslab.org, accessed on 27 June 2024) and STP (http://www.swisstargetprediction.ch, accessed on 27 June 2024). A total of 625 CRC-related targets were collected in GeneCards (https://www.genecards.org, accessed on 17 July 2024) with the filtering criteria of ‘relevance score > 15’, DisGeNeT (https://www.disgenet.org/search, accessed on 17 July 2024) with ‘score_gda ≥ 0.35’, and CTD (https://ctdbase.org, accessed on 17 July 2024) with ‘inference score > 60’. Venny 2.1.0 (https://bioinfogp.cnb.csic.es/tools/venny, accessed on 17 July 2024) was used to draw a Venn diagram. Cytoscape 3.10.2 software was used to construct a network to connect compounds of *C. sappan* L. with the selected 87 target genes. The STRING database was used to construct the protein and PPI network. Pathway enrichment analysis was performed with the gene sets of GO terms of the BPs, CCs, and MFs and the KEGG using the bioinformatics resource DAVID (https://david.ncifcrf.gov, accessed on 24 July 2024).

### 4.5. Cell Culture and CSE Treatment

Human colon cancer HCT 116 and KM12SM cells were obtained from the Korean Cell Line Bank (Seoul, Republic of Korea). Human colon cancer HT-29 and COLO 205 cells were obtained from the American Type Culture Collection (Manassas, VA, USA). Murine colon cancer MC38 cells were obtained from ABM (Richmond, BC, Canada). HCT 116, KM12SM, HT-29, and COLO 205 cells were grown in RPMI medium, and MC38 cells were grown in DMEM, supplemented with 10% heat-inactivated FBS, 100 units/mL penicillin, and 100 μg/mL streptomycin. Cells were maintained at 37 °C in a humidified atmosphere with 5% CO_2_. Cells were treated with various concentrations of CSE for 24 or 48 h. In all cell experiments, the concentration of dimethyl sulfoxide was maintained at 0.03%.

### 4.6. Cell Proliferation Assay

HCT 116, KM12SM, HT-29, and COLO 205 cells (1 × 10^4^ cells/well) were seeded onto a 96-well plate. The next day, cells were treated with various concentrations of CSE for 48 h. Cell proliferation was determined by measuring the cellular ATP levels using CellTiter-Glo 2.0 (Promega, Madison, WI, USA) and dehydrogenase activities using Cell Counting Kit-8 (CCK-8, Dojindo, Tokyo, Japan). The luminescence of the CellTiter-Glo reagent and the absorbance of the CCK-8 reagent (450 nm) were recorded by a Spectramax i3 microplate reader (Molecular Devices, San Jose, CA, USA). Dose–response curves were generated to calculate the IC_50_ values of CSE using GraphPad Prism 9 (San Diego, CA, USA).

### 4.7. Analysis of Cell Apoptosis by Flow Cytometry

Cell apoptosis was performed by flow cytometry. Briefly, HCT 116 and COLO 205 cells were seeded at a density of 5 × 10^5^ cells/well onto a 6-well plate and incubated overnight. Various concentrations of CSE were treated with CSE for 24 and 48 h. Subsequently, cells were stained with Annexin V-FITC and 7-AAD for 15 min in the dark. The cells were detected using a BD LSRFortessa™ X-20 flow cytometer (BD Biosciences, San Diego, CA, USA). The rate of Annexin V-FITC-positive cells, which means the apoptotic rate, was analyzed using Flowjo 10.0 software (BD Biosciences).

### 4.8. Measurement of Caspase-3/7 Activity

The induction of apoptosis was evaluated by measuring the caspase-3/7 activity with the Caspase-Glo 3/7 Assay Kit (Promega). Briefly, HCT 116 and COLO 205 cells were seeded at a density of 1 × 10^4^ cells/well onto 96-well plates and allowed to adhere overnight. The cells were treated with various concentrations of CSE for 48 h. Caspase-3/7 reagent was added to each well and incubated for 1 h at room temperature in the dark. The luminescence was measured using a Spectramax i3 microplate reader. The data were expressed as fold change to the untreated cells.

### 4.9. Protein Extraction and Proteome Array Analysis

HCT-116 and COLO 205 cells were seeded at a density of 5 × 10^5^ cells/well onto a 6-well plate and incubated overnight. The cells were exposed to various concentrations of CSE for 24 and 48 h. Cell lysates were prepared using Cell Lysis Buffer II (Invitrogen, Carlsbad, CA, USA), supplemented with Halt™ Protease and Phosphatase Inhibitor Cocktail (Thermo Fisher Scientific, Waltham, MA, USA). Protein concentrations were determined with a Qubit™ Protein Assay Kit (Invitrogen). The Human Apoptosis Proteome Profiler Antibody Array kit (R&D Systems, Minneapolis, MN, USA) was used to simultaneously confirm the relative expression levels of multiple apoptosis-related proteins from HCT 116 and COLO 205 cells after CSE treatment (30 μg/mL). The blots were developed using Clarity ECL Western Blotting Substrates (Bio-Rad, Hercules, CA, USA) and visualized using an ImageQuant LAS 4000 mini (GE Healthcare, Chicago, IL, USA).

### 4.10. Western Blotting

The protein samples were prepared in 4× LDS sample buffer (Invitrogen) and 10× sample reducing agent (Invitrogen) and then heated at 75 °C for 10 min. The samples were separated on 10 or 12% Bis-Tris gels in MOPS SDS running buffer and transferred onto a 0.2 μm nitrocellulose membrane (Invitrogen) using the SureLock Tandem Transfer System (Thermo Fisher Scientific). The membrane was blocked with EzBlock Chemi (ATTO, Tokyo, Japan), incubated with primary antibodies overnight, and finally incubated with secondary antibodies conjugated with horseradish peroxidase. The bands were developed using EzWestLumi Plus (ATTO) and visualized using an ImageQuant LAS 4000 mini.

### 4.11. Quantitative RT-PCR (qRT-PCR)

HCT 116 cells were seeded at a density of 5 × 10^5^ cells/well onto a 6-well plate and incubated overnight. Total RNA was extracted using the RNeasy Plus Mini Kit (Qiagen, Valencia, CA, USA). The purity and RNA concentration were measured using Nanodrop (Thermo Scientific, Waltham, MA, USA), and 1 μg of RNA was converted to cDNA using the iScript™ Advanced cDNA Synthesis kit (Bio-Rad). The Human Cancer Inflammation & Immunity Crosstalk RT^2^ Profiler PCR Array (PAHS-181Z, Qiagen) was used to profile the expression of 84 key genes. The sequence information of the primers used is shown in Table S1. Gene expression analysis was performed using SsoAdvanced SYBR Green Supermix kit (Bio-Rad) on a Bio-Rad CFX Connect Real-Time System (Bio-Rad) under the following reaction conditions: initial denaturation and enzyme activation at 95 °C for 2 min, followed by 40 cycles of amplification at 95 °C for 5 s and 60 °C for 30 s. Gene expression levels were calculated using the 2^−ΔΔCt^ method and normalized by the housekeeping gene ACTB.

### 4.12. Tumor-Bearing Mice and Treatment

Specific-pathogen-free six-week-old female C57BL/6 mice were purchased from Saeron Bio (Uiwang, Republic of Korea) and acclimated for one week before experimental use. The mice were housed under the following environmental conditions: temperature, 23 °C; humidity, 50%; 12 h light/dark cycle. Animals were fed a standard chow diet (Purina Co., Seoul, Republic of Korea) and provided drinking water ad libitum. All experimental procedures were approved by the Animal Care and Use Committee of the Korea Institute of Oriental Medicine (Approval number: 22-054). MC38 cell tumor-bearing mice were established by subcutaneously inoculating 1 × 10^5^ MC38 cells into the right flank of C57BL/6 mice. The mice were divided into two groups (n = 8/group) when the tumor volume reached >60 mm^3^. CSE was prepared by dissolving it in 1% CMC in DW at a concentration of 20 mg/mL. The mice were administrated 100 mg/kg of CSE daily via oral gavage, while control mice received the same volume of 1% CMC in DW. Tumor volume was measured every 2 to 3 days using calipers and determined by the following formula: ½ × length × width^2^. The body weight of mice was monitored during the experiments.

### 4.13. Hematoxylin and Eosin Staining

Animal tissues were fixed in 10% neutral-buffered formalin (Sigma-Aldrich, St. Louis, MO, USA) for 2 days and processed into paraffin blocks. Paraffin-embedded sections (4 μm thick) were stained with hematoxylin and eosin. The stained slides were cover-slipped using Leica MM24 mounting medium (Leica Biosystems, Richmond, IL, USA) and scanned using Pannoramic DESK (3DHistech, Budapest, Hungary).

### 4.14. Differential Gene Expression (DEG) Analysis

RNA sequencing data were processed to identify differentially expressed genes (DEGs) between CSE-treated and control groups. The RNA for transcriptomic analysis was extracted from a single tumor per treatment group. The raw counts were normalized, and DEG analysis was performed using the DESeq2 package in R. DEGs were determined based on an adjusted *p*-value threshold of <0.05 and a log_2_ fold change (log_2_FC) threshold of ±1. Volcano plots were generated to visualize the DEGs, highlighting significantly upregulated and downregulated genes.

### 4.15. Gene Set Enrichment Analysis (GSEA)

To explore the biological pathways influenced by the DEGs, GSEA was conducted using the fgsea package in R. Enrichment analysis was performed on ranked gene lists to identify significantly enriched pathways with false discovery rate (FDR) < 0.25. Enriched pathways were visualized to assess the involvement of key cellular processes, such as apoptosis, immune response, and cell cycle regulation.

### 4.16. Network Visualization

To investigate the molecular mechanisms and interactions of DEGs, a drug–pathway–gene interaction network was constructed. EnrichR was employed to perform functional enrichment analysis, and Cytoscape was used to visualize the network of upregulated and downregulated DEGs across key pathways. This network provided insights into the complex interactions between CSE-regulated genes and their associated cellular processes.

### 4.17. Statistical Analysis

Statistical analyses were performed using GraphPad Prism 9 software. Data with error bars represent the mean ± standard deviation (SD), except for tumor volume, which is represented by the standard error of the mean (SEM). Statistical analysis of significance was based on a two-tailed Student’s *t*-test. A value of *p* < 0.05 was chosen as the criterion for statistical significance.

## 5. Conclusions

Our study provides compelling evidence for the multi-target anticancer potential of CSE in treating CRC. By combining network pharmacology and experimental approaches, we have enhanced the understanding of how CSE interacts with CRC-specific cellular processes, which may include the modulation of apoptotic and proliferative pathways. Further studies are needed to translate these findings into clinical applications, but the therapeutic promise of natural products like CSE remains strong and may pave the way for innovative treatments in oncology.

## Figures and Tables

**Table 1:** Qualitative analysis and tentative identification of chemical components in CSE by UHPLC-QTOF MS/MS-based chemical profile analysis.

No.	Name	Formula	Mass (Da)	RT (min)	Adduct	Found at Mass (Da)	Error (ppm)	MS/MS Product Ions	Identified with
1	3′-O-Methylcatechin	C_16_H_16_O_6_	304.0947	5.69	[M + H]^+^	305.1019	−0.3	123.0448	*
					[M − H]^−^	303.0872	−0.8	148.0164, 163.0399, 123.0460, 161.0242, 162.0325	
2	Brazilin	C_16_H_14_O_5_	286.0841	6.42	[M + H]^+^	287.0917	1	131.0483, 149.0587, 103.0540, 177.0529	#
					[M − H]^−^	285.0769	0.2	163.0406, 135.0456, 121.0301, 267.0672	
3	Protosappanin B	C_16_H_16_O_6_	304.0947	6.65	[M + H]^+^	305.1020	−0.1	229.0491, 213.0557, 239.0714, 203.0706	*
					[M − H]^−^	303.0875	0.3	231.0667, 230.0586, 243.0668, 255.0665	
4	Chrysophanol	C_15_H_10_O_4_	254.0579	6.66	[M + H]^+^	255.0652	−0.1	153.0691	*
5	Dihydroxyxanthone	C_13_H_8_O_4_	228.0423	6.67	[M + H]^+^	229.0495	−0.3	155.0455, 173.0586, 183.0477	*
6	4′-O-Methylcatechin	C_16_H_16_O_6_	304.0947	7.26	[M − H]^−^	303.0875	0.2	163.0400, 135.0452, 162.0332, 121.0296, 148.0170	*
7	Lyoniresinol	C_22_H_28_O_8_	420.1784	8.62	[M + H]^+^	421.1860	0.8	249.1129	*
					[M − H]^−^	419.1703	−1.9	373.1279, 359.1226, 283.0571	
8	3′-O-Methylbrazilin	C_17_H_16_O_5_	300.0998	8.93	[M + H]^+^	301.1069	−0.6	167.0329, 123.0437, 93.0337, 255.0630	*
					[M − H]^−^	299.0923	−0.7	163.0401, 135.0452, 145.0294, 281.0454	
9	Brazilein	C_16_H_12_O_5_	284.0685	8.99	[M + H]^+^	285.0760	0.8	147.0439, 175.0386, 239.0703, 165.0703	#
					[M − H]^−^	283.0614	0.6	265.0508, 196.0525, 240.0425, 173.0240	
10	Sappanone B	C_16_H_14_O_6_	286.0841	9.05	[M + H]^+^	303.0865	0.6	123.0438, 163.0384, 137.0222	*
					[M − H]^−^	301.0720	0.8	151.0399, 179.0350, 177.0201, 135.0091	
11	Sappanchalcone	C_16_H_14_O_5_	286.0841	10.92	[M + H]^+^	287.0917	0.9	151.0389, 108.0209, 65.0408	*
					[M − H]^−^	285.0770	0.4	134.0374, 161.0242, 148.0163, 269.0454, 133.0295	
12	5,7,4′-Tri-O-methylcatechin	C_18_H_20_O_6_	332.1260	11.2	[M + H]^+^	333.1331	−0.4	No MS/MS	*
					[M − H]^−^	331.1185	−0.8	148.0170, 163.0170, 135.0458, 121.0304	
13	Sappanone A	C_16_H_12_O_5_	284.0685	11.34	[M + H]^+^	285.0756	−0.6	175.0399, 147.0440, 239.0697	*
					[M − H]^−^	283.0612	0	147.0450, 265.0508, 135.0096, 161.0238	
14	5,7,3′-Tri-O-methyl (-)-epicatechin	C_18_H_20_O_6_	332.1260	11.94	[M + H]^+^	333.1334	0.3	123.0436, 159.0423, 138.0675, 77.0379	*
					[M − H]^−^	331.1190	0.7	163.0407, 148.0174, 135.0461, 162.0331	
15	Brazilane	C_16_H_14_O_4_	270.0892	12.41	[M + H]^+^	271.0966	0.4	151.0388, 108.0217, 147.0442	*
					[M − H]^−^	269.0822	0.9	253.0506, 225.0552, 161.0242, 145.0296	
16	10,11-Dihydroxydracaenone C	C_16_H_14_O_4_	270.0892	13.09	[M − H]^−^	269.0819	0	147.0454, 109.0310, 159.0441, 129.0350	*
17	4′-O-Methylbrazilin	C_17_H_16_O_5_	300.0998	13.25	[M + H]^+^	301.1072	0.4	131.0487, 163.0749, 103.0547	*
					[M − H]^−^	299.0923	−0.7	163.0416, 135.0454	
18	Tricin	C_17_H_14_O_7_	330.0740	16.08	[M + H]^+^	331.0815	0.9	316.0579, 315.0512, 273.0394, 98.9851	#
					[M − H]^−^	329.0669	0.7	299.0193, 314.0432, 271.0242, 243.0279	

# In-house MS/MS library and online databases, such as GNPS, MASS bank, or Metlin. * Formula from literature based on Extract MS and isotope mass pattern.

## Data Availability

The original contributions presented in the study are included in the article/Supplementary Materials; further inquiries can be directed to the corresponding author.

## Supplementary Materials

The following supporting information can be downloaded at https://www.mdpi.com/article/10.3390/plants14020263/s1: Figure S1: MS/MS spectra of qualitative analysis and tentative identification of chemical components in CSE by UHPLC-QTOF MS/MS-based chemical profile analysis; Figure S2: The higher resolution of Figure 2B,C; Figure S3: The higher resolution of Figure 3E; Figure S4: The higher resolution of Figure 10A,D; Table S1: The sequences of the primers used in qRT-PCR.
