# Supplementary material for: An Investigation of the Anticancer Mechanism of Caesalpinia sappan L. Extract Against Colorectal Cancer by Integrating a Network Pharmacological Analysis and Experimental Validation
Source: Plants (Basel). 2025 Jan 18;14(2):263. doi: 10.3390/plants14020263 (PMC11768342; doi:10.3390/plants14020263)
Supplement: Supplementary file 1 [file plants-14-00263-s001.zip › Supplementary Materials.pdf]

plants

MDPI

Supplementary Materials

Figure S1. MS/MS spectra of qualitative analysis and tentative identification of chemical components in CSE by UHPLC-QTOF MS/MS-based chemical profile analysis.

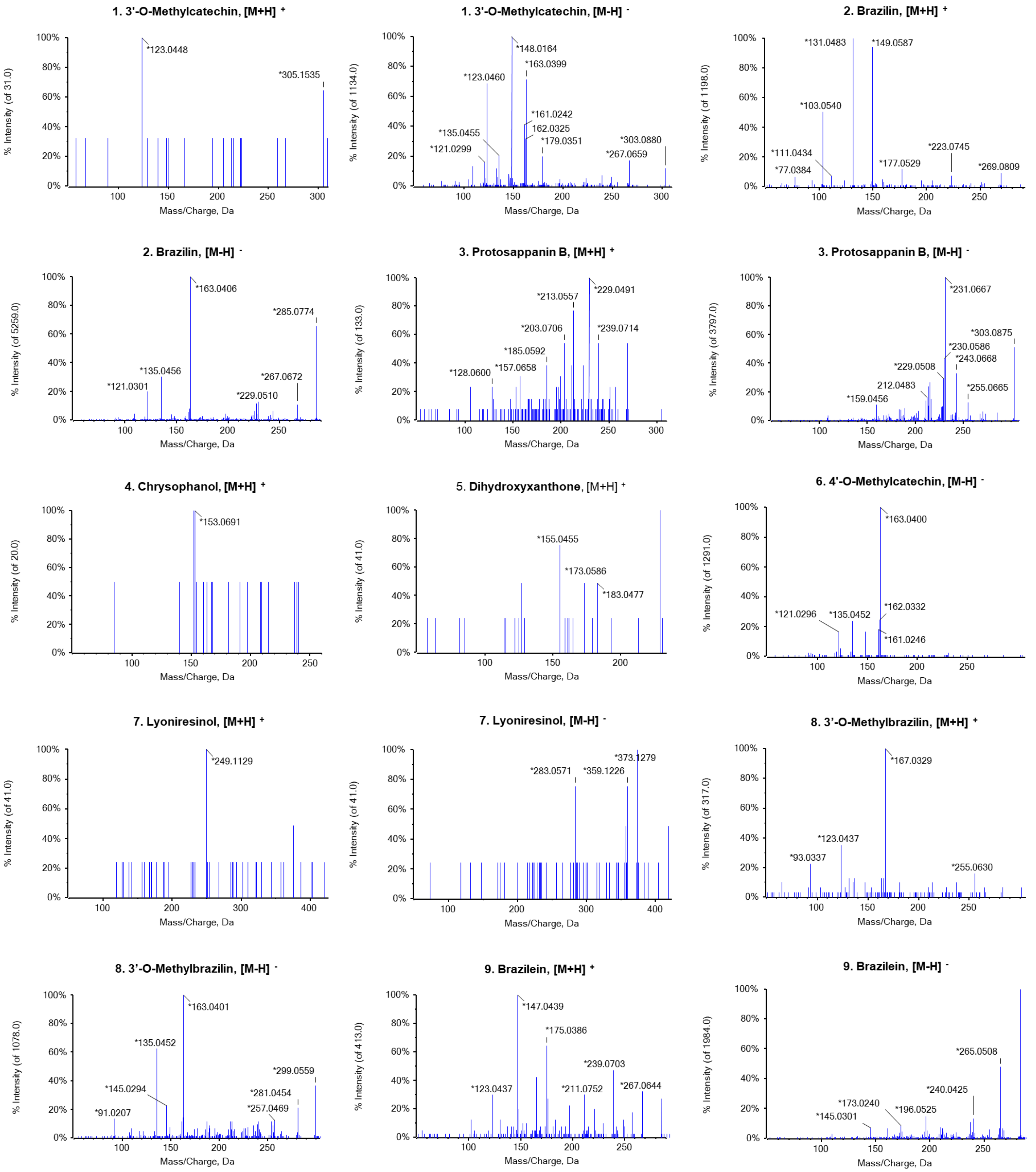

10.Sappanone B, [M+H] +

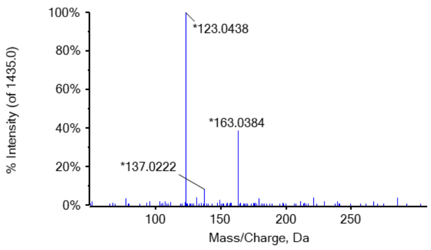

10.Sappanone B, [M-H] -

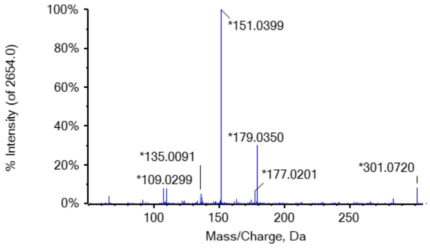

11. Sappanchalcone, [M+H] +

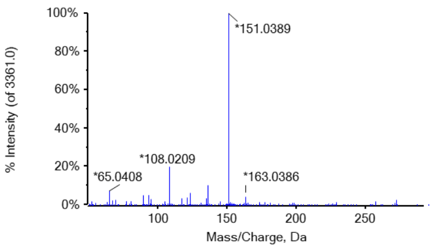

11. Sappanchalcone, [M-H] -

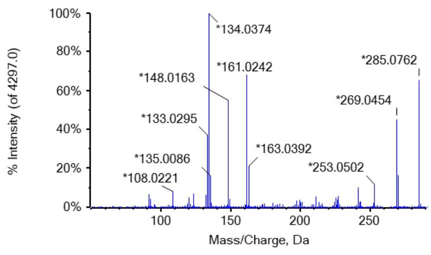

12. 5,7,4'-Tri-O-methylcatechin, [M-H] -

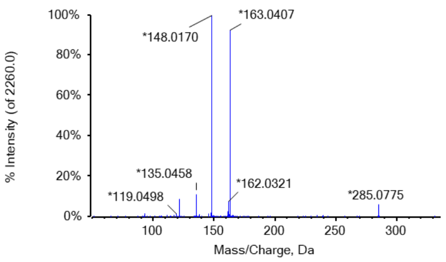

13. Sappanone A, [M+H] +

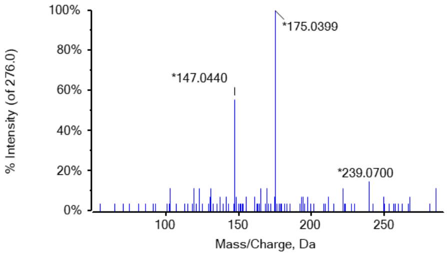

13. Sappanone A, [M-H] -

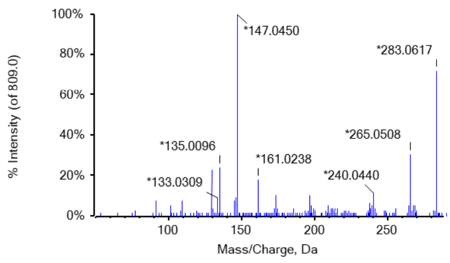

14. 5,7,3'-Tri-O-methyl (-)-epicatechin, [M+H] +

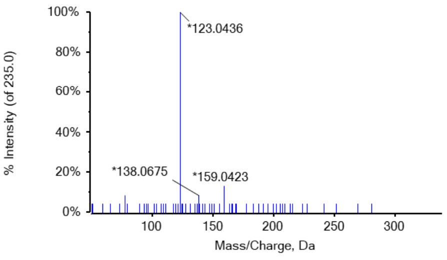

14. 5,7,3'-Tri-O-methyl (-)-epicatechin, [M-H] -

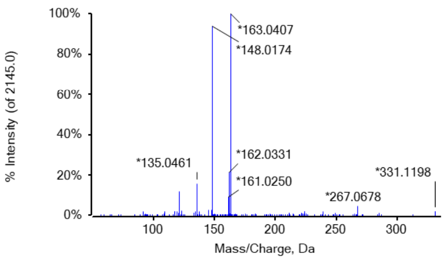

15. Brazilane, [M+H] +

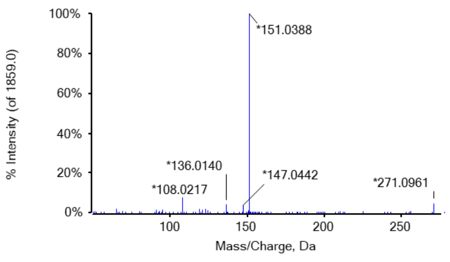

15. Brazilane, [M-H] -

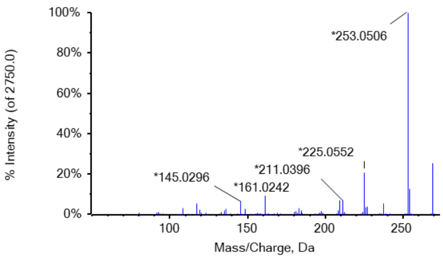

16. 10,11-Dihydroxydracaenone C, [M+H] +

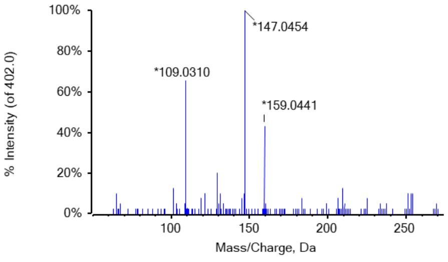

17. 4'-O-Methylbrazilin, [M+H] +

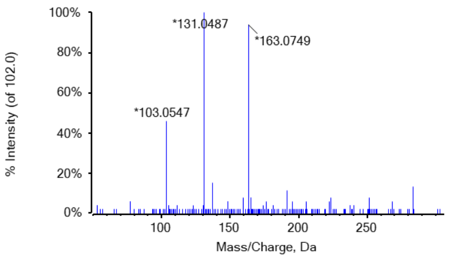

17. 4'-O-Methylbrazilin, [M-H] -

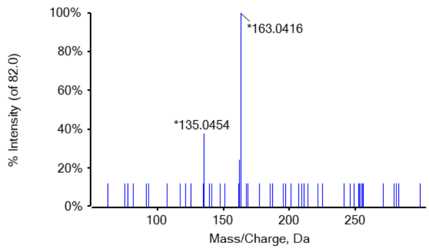

18. Tricin, [M+H] +

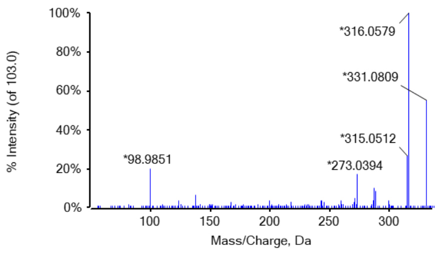

18. Tricin , [M-H] -

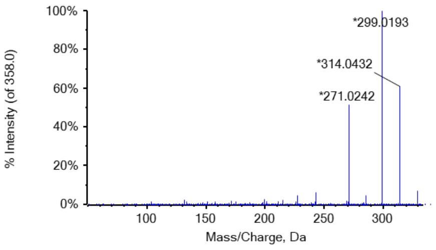

Figure S2. The higher resolution of Figure 2B and C. (**A**) Network of compounds and target genes of CSE. (**B**) Protein and protein interaction (PPI) of the common 87 target genes.

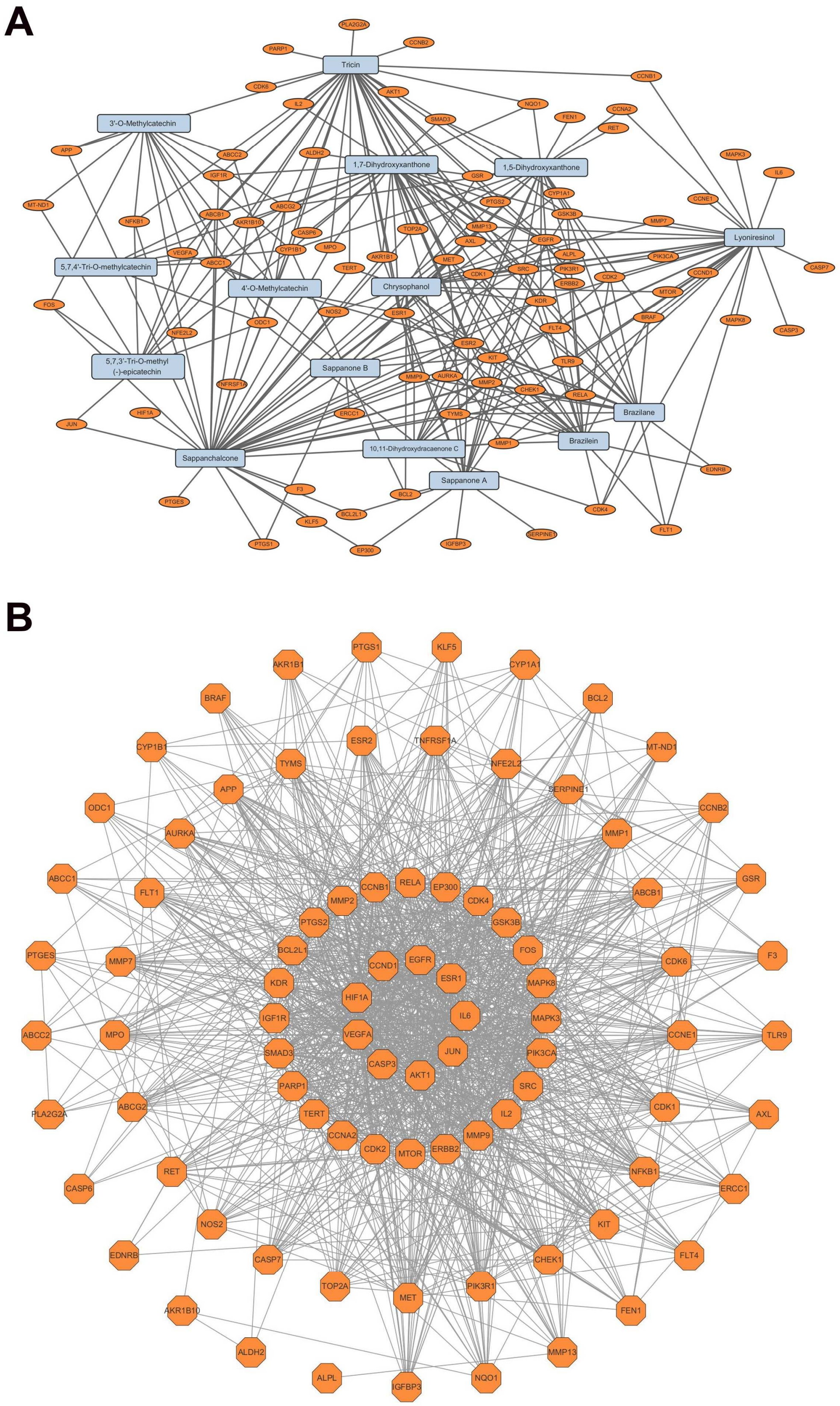

Figure S3. The higher resolution of Figure 3E. Pathways in cancer involved in cell cycle regulation, apoptosis, and signal transduction.

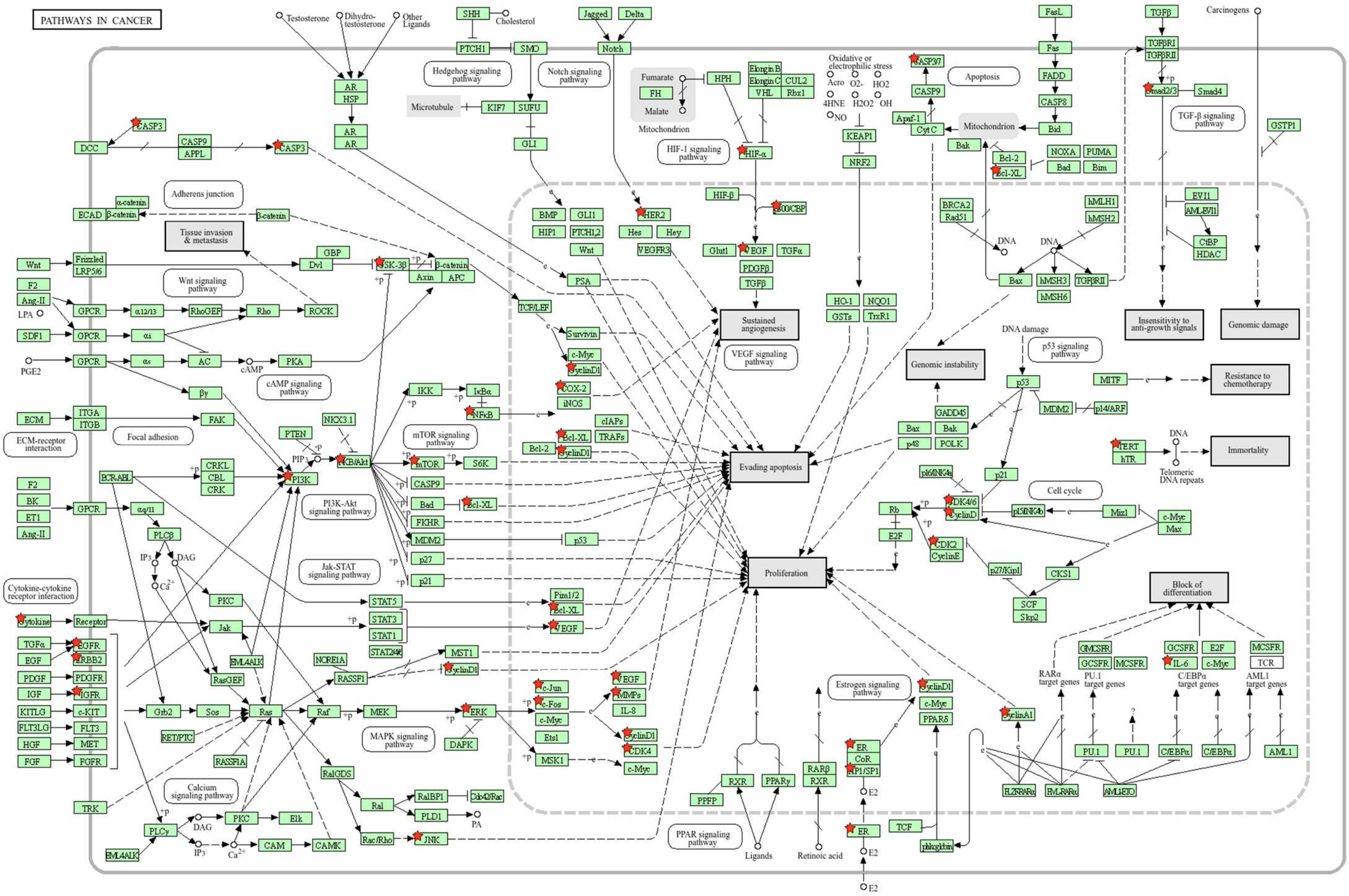

Figure S4. The higher resolution of Figure 4A and D. (**A**) Volcano plot depicting differentially expressed genes (DEGs) between CSE-treated and control groups. (**B**) Drug-pathway-gene interaction network reconstructed using the GSEA results.

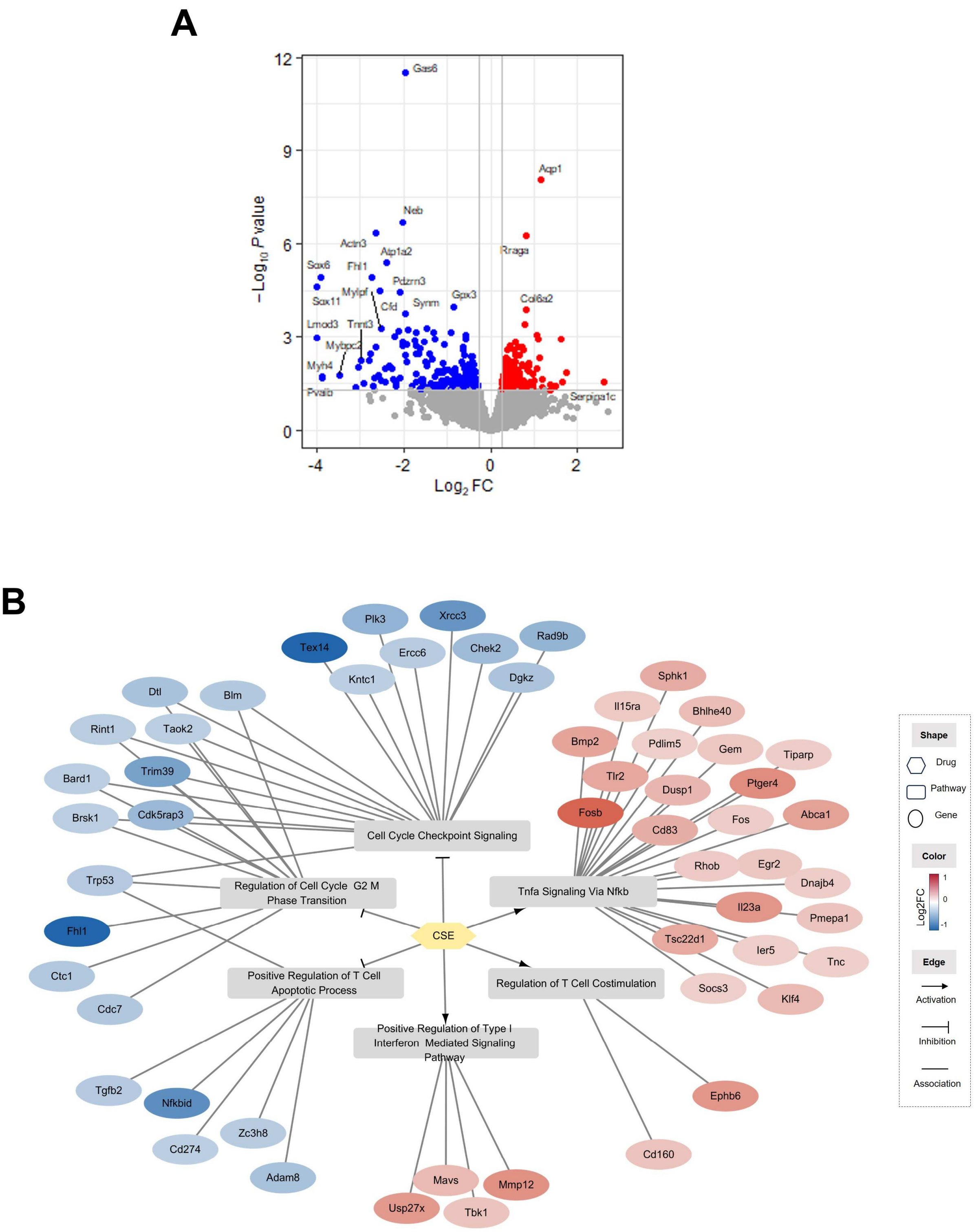

Table S1. The sequences of the primers used in qRT-PCR.

Gene	Forward (5′-3′)	Reverse (5′-3′)
CCND2	GAG AAG CTG TCT CTG ATC CGC A	CTT CCA GTT GCG ATC ATC GAC G
CCND1	TCT ACA CCG ACA ACT CCA TCC G	TCT GGC ATT TTG GAG AGG AAG TG
TRADD	ACA AGG TGG TCC TGT CGG ATG C	CGG TGG ATC TTC AGC ATC TGC A
CCNE1	TGT GTC CTG GAT GTT GAC TGC C	CTC TAT GTC GCA CCA CTG ATA CC
CDKN2A	CTC GTG CTG ATG CTA CTG AGG A	GGT CGG CGC AGT TGG GCT CC
CDKN2B	ACG GAG TCA ACC GTT TCG GGA G	GGT CGG GTG AGA GTG GCA GG
CDKN1A	AGG TGG ACC TGG AGA CTC TCA G	TCC TCT TGG AGA AGA TCA GCC G
CDKN3	ATG GAG GGA CTC CTG ACA TAG C	TCT CCC AAG TCC TCC ATA GCA G
CDK6	GGA TAA AGT TCC AGA GCC TGG AG	GCG ATG CAC TAC TCG GTG TGA A
CCNB2	CAA CCA GAG CAG CAC AAG TAG C	GGA GCC AAC TTT TCC ATC TGT AC
CCNB1	GAC CTG TGT CAG GCT TTC TCT G	GGT ATT TTG GTC TGA CTG CTT GC
CDC20	TAG TTG CCC TCA AGA AGG TGG C	GTG TGG GAA CAC AGC CTT CAG T
CDKN1B	ATA AGG AAG CGA CCT GCA ACC G	TTC TTG GGC GTC TGC TCC ACA G
MMP9	GCC ACT ACT GTG CCT TTG AGT C	CCC TCA GAG AAT CGC CAG TAC T
CDK1	GGA AAC CAG GAA GCC TAG CAT C	GGA TGA TTC AGT GCC ATT TTG CC
CCNA2	CTC TAC ACA GTC ACG GGA CAA AG	CTG TGG TGC TTT GAG GTA GGT C
FADD	CTC CTG CGC GAG CTG CTC GC	GCC TTC TCC AAT CTT TCC CCA C
CDC16	GTG TCT TGG TTT GCA GTG GGA TG	GTG CTC ACT CTC CAC CGC AAA T
CDK4	CCA TCA GCA CAG TTC GTG AGG T	TCA GTT CGG GAT GTG GCA CAG A
FAS	GGA CCC AGA ATA CCA AGT GCA G	GTT GCT GGT GAG TGT GCA TTC C
CDK2	ATG GAT GCC TCT GCT CTC ACT G	CCC GAT GAG AAT GGC AGA AAG C
CKS1	GGA ATC TTG GCG TTC AGC AGA G	GAG GCT GAA AAG TAG CTT GCC AG
CKS2	GAG GAG ACT TGG TGT CCA ACA G	GAT TTG ACG ATC CCC AGA TAA ACT
TNFR1	CCG CTT CAG AAA ACC ACC TCA G	ATG CCG GTA CTG GTT CTT CCT G
MMP2	AGC GAG TGG ATG CCG CCT TTA A	CAT TCC AGG CAT CTG CGA TGA G
